# Associations between Advanced Glycation End Products, Body Composition and Mediterranean Diet Adherence in Kidney Transplant Recipients

**DOI:** 10.3390/ijerph191711060

**Published:** 2022-09-04

**Authors:** Josipa Radić, Marijana Vučković, Andrea Gelemanović, Ela Kolak, Dora Bučan Nenadić, Mirna Begović, Mislav Radić

**Affiliations:** 1 Department of Nephrology and Dialysis, University Hospital of Split, Spinčićeva 1, 21 000 Split, Croatia; 2 Department of Internal Medicine, University of Split School of Medicine, Šoltanska 2, 21 000 Split, Croatia; 3 Mediterranean Institute for Life Sciences (MedILS), 21 000 Split, Croatia; 4 Department of Nutrition and Dietetics, University Hospital Centre Split, 21 000 Split, Croatia; 5 School of Medicine, University of Split, Šoltanska 2, 21 000 Split, Croatia; 6 Division of Clinical Immunology and Rheumatology, Department of Internal Medicine, University Hospital of Split, 21 000 Split, Croatia

**Keywords:** advanced glycation end products, Mediterranean diet, kidney transplant, body composition

## Abstract

There is limited evidence on the associations between dietary patterns, body composition, and nonclassical predictors of worse outcomes such as advanced glycation end products (AGE) in kidney transplant recipients (KTRs). The aim of this cross-sectional study was to determine the level of AGE-determined cardiovascular (CV) risk in Dalmatian KTRs and possible associations between AGE, adherence to the Mediterranean diet (MeDi), and nutritional status. Eighty-five (85) KTRs were enrolled in this study. For each study participant, data were collected on the level of AGE, as measured by skin autofluorescence (SAF), Mediterranean Diet Serving Score (MDSS), body mass composition, anthropometric parameters, and clinical and laboratory parameters. Only 11.76% of the participants were adherent to the MeDi. Sixty-nine percent (69%) of KTRs had severe CV risk based on AGE, while 31% of KTRs had mild to moderate CV risk. The results of the LASSO regression analysis showed that age, dialysis type, dialysis vintage, presence of CV and chronic kidney disease, C- reactive protein level, urate level, percentage of muscle mass, and adherence to recommendations for nuts, meat, and sweets were identified as positive predictors of AGE. The negative predictors for AGE were calcium, phosphate, cereal adherence according to the MeDi, and trunk fat mass. These results demonstrate extremely low adherence to the MeDi and high AGE levels related CV risk in Dalmatian KTRs. Lifestyle interventions in terms of CV risk management and adherence to the MeDi of KTRs should be taken into consideration when taking care of this patient population.

## 1. Introduction

Kidney transplant recipients (KTRs) benefit from kidney transplantation (KTX) in several ways, but still this particular group of patients suffers from increased cardiovascular (CV) risk [1]. The multifactorial etiology of CV risk-weight change, immunosuppression, and post-transplant diabetes mellitus are known to be classic risk factors in the KTRs [2,3,4]. In addition, changes in nutritional status, which carries the risk of sarcopenia, malnutrition, and obesity exacerbated by post-transplant weight gain, as well as the side effects of polypharmacy in this population, are associated with worse outcomes [5,6]. Recent studies have found a nonclassical predictor of worse outcomes in this population—advanced glycation end products (AGE)—which have been associated with CV risk, renal graft failure [7,8], and accelerated arteriosclerosis [9]. Although the plasmatic clearance of AGE dramatically improves after successful KTX, its removal from slow-turnover tissues might be difficult to achieve and may contribute to the high CV disease burden in this population of patients [10]. Moreover, circulating levels of AGE are independently associated with the long-term risk of CV mortality in KTRs [8].

KTX is a complex surgical procedure with a variety of potential perioperative complications ranging from infections to pain, a long hospital stays, and a long recovery period with limited mobility that can affect both nutritional status and inflammatory parameters. Recently, various surgical procedures have been investigated, and minimally invasive robotic-assisted KTX has been shown to be a safe technique that results in fewer surgical complications [11,12,13]. This procedure is thought to be particularly beneficial for obese KTRs, who are more prone to surgical site infections during the early post-transplant period [11,14].

The concentration of AGE in the skin, as measured by skin autofluorescence (SAF), has been associated with CV risk [15,16], atherosclerosis [17], and cumulative oxidative stress [18].

Diet plays an important role in the overall health of KTRs and is receiving increasing attention [19]. Several dietary patterns have been studied in KTRs [20], and the Mediterranean diet (MeDi) has been shown to have a positive effect on graft survival [19], development of post-transplantation diabetes mellitus [21], nutritional status [22,23], and depression [24].

There is evidence that in the general population [25], in patients with diabetes mellitus [26] and premenopausal women [27], lower AGE values correlate with MeDi adherence. In addition, correlations between AGE value, fat mass [28], and muscle mass [29] were found in the general population.

AGE molecules are formed during the Maillard reaction [30,31], and ⅔-consumed AGE accumulates in the body. As for the dietary sources of AGE, foods rich in AGE are mainly grilled, fried, roasted, and other foods thermally processed under dry conditions [32,33]. The consumption of red meat with vegetables, spices, herbs, and fruits may reduce AGE [33,34,35,36,37,38,39]. A meta-analysis of 17 randomized controlled trials comparing a low AGE diet with a high AGE diet suggests that a low AGE diet may reduce cardiometabolic disease risk markers such as insulin, low-density lipoprotein (LDL) cholesterol, C-reactive protein (CRP), and adhesion molecules, as well as inflammatory markers [33,40]. A review by Bettiga found associations between obesity, AGE, and the early development of kidney disease [41].

To our knowledge, there are no studies that examined the association between nutritional status, MeDi adherence, and AGE in KTR. Therefore, the aim of this study was to investigate the levels of AGE in Dalmatian KTRs and possible associations between AGE, MeDi adherence, and nutritional status in this population of patients.

## 2. Materials and Methods

Eighty-five KTRs, with a mean age of 63, and of which 58.82% were male, with a mean time since the KTX being 8 years, were included in this cross-sectional study conducted at the outpatient clinic of the Department of Nephrology and Dialysis, University Hospital of Split, Croatia.

The study protocol was approved by the Ethics Committee of the University Hospital of Split, Croatia.

A detailed protocol is described in our previous study [22], which included 155 KTRs, out of which we excluded 56 patients due to a lack of AGE measurement, 6 patients without CV risk based on AGE, 6 patients that were not on dialysis treatment before KTX, and 2 patients with incomplete MDSS questionnaires.

### 2.1. Body Composition and Anthropometric Measurement

Body composition was assessed using an MC-780 Multi Frequency Segmental Body Analyzer (Tanita, Tokyo, Japan) for each study participant. The device uses a constant high-frequency current flow and eight electrodes to determine the electrical resistance of different tissues. The method is called bioelectrical impedance analysis (BIA). It is used to assess fat mass (kg), fat mass percentage (%), fat-free mass (kg), visceral fat level, muscle mass (kg), skeletal muscle mass (kg), skeletal muscle mass percentage (%), body mass (kg), phase angle (°) and trunk fat mass (kg). All of the patients were advised not to ingest any food or liquid at least 3 h before the measurement, to urinate just before the measurement, and not to consume alcohol, eat, or drink excessively, or exercise in an excessive way for at least one day before the body composition measurement [42]. Height was measured using a stadiometer. Waist circumference (WC) and mid-upper arm circumference (MUAC) were measured using a flexible, non-stretchable measuring tape in a standing position facing forward with their shoulders relaxed. Body mass index (BMI) and waist-to-height ratio (WHtR) were calculated for each study participant.

### 2.2. Mediterranean Diet Serving Score

The validated Mediterranean Diet Serving Score (MDSS) questionnaire was used to determine adherence to the MeDi, considering the consumption of different foods and food groups (MeDi components) in time intervals per meal, day or week [43].

The food was divided into fourteen food groups, and the points were given according to the new Mediterranean food pyramid in the following way: three points for fruits, vegetables, olive oil, and cereals if consumed with each meal; two points for dairy products and nuts if consumed daily; one point for the recommended number of servings per week is consumed for potatoes (≤3), legumes (≥2), eggs (2–4), fish (≥2), white meat (2), red meat (<2), sweets (≤2), and fermented beverages (1–2 glasses a day) [44].

It sums up to a maximum MDSS score of twenty-four (24), and the greater score implies greater adherence to the MeDi. The optimal cut-off point ≥13.50 was set to determine adherence or non-adherence to the MeDi [43].

### 2.3. Medical History, Clinical and Laboratory Parameters

By a thorough examination of patient’s medical records, data concerning the existence and duration of primary chronic kidney disease (CKD), arterial hypertension, diabetes mellitus, and CV events, as well as the time of KTX, the type and duration of dialysis treatment before KTX were provided.

Regarding laboratory parameters, all the study participants underwent the usual peripheral blood sampling for the purpose of the study, which was collected by the trained project nurse, and they were asked to obtain a 24-h urine sample on the same day as the body composition measurement, AGE measurement, MeDi adherence, and blood pressure measurement. Blood pressure was measured using a sphygmomanometer Omron, Kyoto, Japan) on the left and right upper arms, and 3 consecutive measurements with a 1 min distance were performed on the arm on which a higher level of blood pressure was detected. The mean value was calculated. We collected data on the levels of urea (mmol/L), creatinine (mmol/L), uric acid (mmol/L), serum albumin (g/L), phosphates (mmol/L), CRP (mg/L), calcium (mmol/L), glucose (mmol/L), triglycerides (mmol/L), total cholesterol (mmol/L), LDL cholesterol (mmol/L), haemoglobin (g/L), mean cellular volume (MCV), sodium (mmol/L), potassium (mmol/L), and estimated glomerular filtration rate (eGFR) using Chronic Kidney Disease Epidemiology Collaboration (CKD-EPI) equation (mL/min/1.73 m^2^). The blood samples for the analysis of the serum levels of complement components were collected in standard test tubes without additives in the Laboratory of Medical Diagnostics and Biochemistry at the University Hospital of Split, Croatia, and were centrifuged at 1690× *g* on the HERMLE Z400 centrifuge model (Hermle Labortechnik GmbH, Wehingen, Germany). The concentrations of urea (mmol/L), uric acid (mmol/L), serum albumin (g/L), phosphates (mmol/L), and CRP (mg/L) were determined via standard laboratory methods. For the creatinine measurement, the Jaffe method was used. A complete blood count was obtained using a hematology analyzer (Advia 120, Siemens, Erlangen, Germany).

### 2.4. Advanced Glycation End Products (AGE) Measurement

AGEs were measured on the skin using SAF, a noninvasive desktop device (AGE Reader mu, Diagnostic’s Technologies BV, Groningen, The Netherlands). SAF is expressed in arbitrary units (AU) [45].

The device uses a UV-A light-emitting lamp and a built-in spectrometer to calculate SAF by dividing the excitation light by the emitted light. Before measurement, the skin of a participant’s dominant forearm was cleaned with alcohol and placed on the device. All the measurements were performed on a skin site that had no visible abnormalities. Three consecutive measurements were taken for each study participant, and the mean value of SAF was calculated. The measured SAF level was considered together with the age of the participants, and a score was calculated that classified the participants into one of the four groups (none, limited, increased, definite) according to their CV risk [46] using an application provided by the manufacturer. Four groups were then merged into two groups for statistical analysis where none and limited were categorized as mild to moderate CV risk and increased and definite were categorized as severe CV risk.

### 2.5. Statistical Analyses

After the exclusion of the participants who had missing data, the normality of the variables was checked with the Shapiro–Wilk test. The data were described with median and interquartile range (IQR) or with mean and standard deviation (SD) based on normality or as numbers with percentages for categorical variables. To test if the two groups of participants differed based on AGE severity, descriptive statistics were performed first using the Mann–Whitney test, t-test, or chi-square test where appropriate. To find the predictors of severe AGE, first, univariate logistic (mild and moderate vs. severe AGE) and linear (AGE score) regression analyses were performed. All the variables with a *p*-value < 0.2 from the univariate regression models were used as input for the LASSO regression models with 10-fold cross-validation. LASSO regression is a modification of a normal regression with penalization, which performs better in situations with smaller sample sizes and where a chance for multicollinearity issues is high. Due to the shrinkage of unimportant coefficients to zero, in LASSO regression, overfitting is avoided, and the model automates feature selection. Univariate regression models are reported with an odds ratio (OR) and 95% confidence interval (95% CI) or beta (β) with standard errors (SE) as appropriate, while LASSO regression models are reported with LASSO coefficients and 95% confidence intervals. *p*-values < 0.05 were considered to be statistically significant. All of the statistical analyses were performed using the free software environment for statistical computing R version 4.0.0 [47]. LASSO regression models were performed with R package glmnet version 4.0-2 [48], while post-selection inference (confidence intervals and *p*-values for the LASSO coefficient) was performed with R package selectiveInference version 4.0.5. [49].

## 3. Results

**Figure 1:**
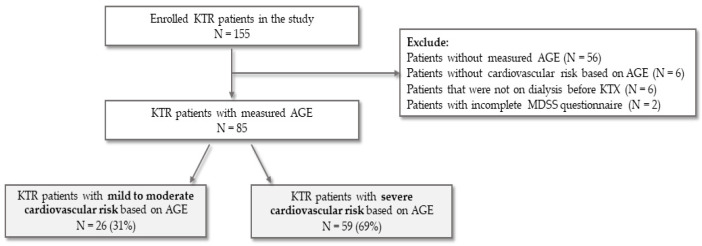
Flowchart of the study protocol.

After the exclusion of KTRs that were not eligible for this study, as shown in Figure 1, a total of 85 KTRs remained for further analysis. Of those, 26 (31%) were categorized as having mild to moderate (AGE median ± IQR = 2.6 ± 0.28), while 59 (69%) were classified as having a severe (AGE median ± IQR = 3.6 ± 0.95) CV risk based on the AGE value.

Abbreviations: KTRs- kidney transplant recipient, AGE- advanced glycation end products, KTX- kidney transplantation, MDSS- Mediterranean Diet Serving Score, N- number.

The general characteristics of these patients are summarized in Table 1. In short, KTRs with severe CV risk based on AGE were significantly older (67 ± 12 years vs. 54 ± 28.5 years; *p* < 0.001), and more of them were treated with hemodialysis (HD) or combined peritoneal dialysis (PD) and HD prior KTX (80% vs. 42%; *p* = 0.003), and more of them suffered from CKD (81% vs. 46%; *p* = 0.002). Differences in smoking status according to CV risk categories did not reach a significant level. Two groups of KTRs did not differ in any of the measured anthropometric parameters and based on the examined laboratory parameters, only differed in the levels of urea and eGFR, where KTRs with severe CV risk had increased levels of urea (10.8 ± 4.85 vs. 8 ± 3.47; *p* = 0.002) and decreased levels of eGFR (39.7 ± 16.55 vs. 61.95 ± 34.43; *p* = 0.009). When looking at the body composition parameters, we observed that KTRs with severe CV risk had significantly higher metabolic age (53.18 ± 11.25 vs. 46.38 ± 16.07; *p* = 0.033) and significantly lower phase angle (4.98 ± 0.76 vs. 5.36 ± 0.67; *p* = 0.036). Finally, two groups of KTRs did not show significantly different patterns in MeDi adherence, as measured through the MDSS questionnaire; in fact, both groups showed very low adherence to the recommended guidelines (only 12% of all KTRs showed MeDi adherence). KTRs suffering from severe CV risk based on the AGE values were slightly more adherent (14% vs. 8%); however, this difference was not statistically significant. When observing each food item or food group, the only significant difference was observed in the pattern of adherence to the recommendations of eating sweets, where KTRs with severe CV risk showed better adherence (66% vs. 35%; *p* = 0.014) (Table 2).

To find the predictors associated with severe CV risk based on the AGE values in KTRs, we first performed univariate logistic and linear regressions to assess how strongly each variable was independently associated with severe CV risk and higher values of AGE, respectively. The results of these univariate regression models are shown in Supplementary Table S1. In both logistic and linear regression, age, the presence of CKD, and levels of urea showed a positive relationship, while levels of eGFR, phase angle, and trunk fat mass showed a negative association with severe CV risk and increased AGE (*p* < 0.05). In addition to that, a positive association with severe CV risk was found with dialysis type, metabolic age, and adherence to sweets recommendations (*p* < 0.05), while an additional positive association with increased AGE levels was found with years spent on dialysis, the presence of CV disease, levels of CRP, levels of urate, and adherence to nuts recommendations (*p* < 0.05). Together with these, all of the variables with *p* < 0.2 were considered potential predictors and were used in further analyses where we employed logistic and linear LASSO regression models. Out of 24 potential predictors, the logistic LASSO regression model selected age (LASSO coefficient 0.098; *p* = 0.01), dialysis type, presence of CKD (LASSO coefficient 1.670; *p* = 0.017), levels of calcium, levels of CRP, levels of urea, skeletal muscle mass, trunk fat mass and adherence to cereals, red meat, and sweets recommendations as important predictors of severe CV risk in KTRs based on AGE (Table 3).

The results of the linear LASSO regression model, of 23 variables input, selected age (LASSO coefficient 0.018; *p* = 0.005), dialysis type, years spent on dialysis, presence of CVD and CKD (LASSO coefficient 0.435; *p* = 0.018), levels of CRP, levels of phosphorus, levels of urate (LASSO coefficient 0.002; *p* = 0.008), trunk fat mass (LASSO coefficient −0.046; *p* = 0.002), and adherence to nuts recommendations were identified as important predictors for increased levels of AGE (Table 3).

The predictors that are retained as statistically significant in both models are highlighted with the LASSO coefficient and *p*-value given in the brackets.

Due to an unexpected negative association between increased AGE and trunk fat mass, we wanted to examine whether this is correlated to the time KTRs have spent on dialysis treatment before KTX or with the time passed since their KTX. The correlation results are shown in Supplementary Figures S1–S8. No significant correlation was found between trunk fat mass and time since KTX or time spent on dialysis before KTX when looking at all 85 KTRs (Figures S1 and S4) or those KTRs that were treated with PD (Figures S5 and S6). A weak negative correlation was found when only taking a subset of the KTRs treated with HD (R = −0.32, *p* = 0.041 for time after KTX (Figure S7); R = −0.29, *p* = 0.067 for time spent on dialysis (Figure S2)), while a very strong positive correlation was found with a subset of KTRs treated with both PD and HD (R = 0.68, *p* = 0.010 for time after KTX (Figure S8); R = 0.69, *p* = 0.011 for time spent on dialysis (Figure S4)).

## 4. Discussion

Our results showed that age and the presence of CKD were the most significant predictors of AGE in KTRs. Moreover, the duration of dialysis treatment before KTX, HD, and the combination of HD/PD were predictive of the AGE values in our studied population. AGE are known predictors of mortality in dialysis patients [50], and in a previous study of KTRs, eGFR was found to be the most important independent determinant of circulating AGE [8]. In a study of 189 stable KTRs, AGE measured by SAF was positively correlated with age, and KTRs with a history of PD showed lower levels of AGE than KTRs with a history of HD [7], which is similar to our results.

The presence of CV events in the history of Dalmatian KTRs was also associated with higher levels of AGE. Previous studies reported that AGE molecules were predictive of CV risk in the general population and in KTRs [51], which is consistent with our results. On the other hand, a study by Calvino et al. found no significant association between the history of CV events and AGE in this population of patients [7]. AGE molecules have been associated with elevated CV risk in patients suffering from CKD. Underlying mechanisms lead to the accumulation of AGE in tissues, which then cross-link with collagen or elastin molecules, resulting in elevated arterial stiffness [52]. SAF-measured AGE was associated with higher carotid intima-media thickness and inversely correlated with endothelial progenitor cells in 212 HD patients, as observed by Ueno et al. [53]

Our results confirm the usefulness of the SAF-measured AGE method, indicating an increased CV risk in this patient population. Regarding the laboratory parameters, our results suggest positive associations between CRP, urate, and urea levels with AGE. Similar results regarding CRP and AGE measured by SAF associations were found in a study by Hartog et al. [54]. A study on type 2 diabetes patients also found positive associations between AGE and uric acid levels [55]. These results suggest the influence of inflammatory and nitrogen excretion products on the levels of AGE.

The results of our final regression model also suggest an inverse relationship between serum calcium and phosphate levels and SAF AGE in KTRs.

Hyperphosphatemia is known to be a predictor of worse outcomes in KTRs [56]. A study of KTRs by Calvino et al. found a positive correlation between SAF and phosphate, which is in contrast to our findings [7]. Phosphate entering vascular smooth muscle cells causes vascular calcification, which in turn increases vascular stiffness and increases the risk of fatal CV events [57]. Higher phosphate levels in the intracellular compartment of vascular smooth muscle cells could explain the inverse relationship we found between serum phosphate levels and AGE.

Calcium is a well-described and extremely important ion involved in the response to various oxidants. It is known that an increase in the level of calcium in the cytoplasm of different cells occurs as a result of different oxidants [58]. This may explain why lower extracellular levels are associated with higher AGE levels in Dalmatian KTRs since AGE molecules are markers of oxidative stress. A study by Calvino et al. found no significant associations between AGE and calcium in KTRs [7].

Regarding body composition, our results are somewhat contradictory. Indeed, our final regression model suggests an inverse relationship between trunk fat mass content and AGE, while at the same time, there is a reciprocal relationship between the percentage of skeletal muscle and AGE in Dalmatian KTRs. A study from Calvino on 189 stable KTRs found negative correlations between SAF-measured AGE and dynamometry [7], and the results from Silva et al. [59] found no associations between AGE and muscle strength in HD patients, while our results suggest differently, suggesting that skeletal muscle mass percentage is a positive predictor of AGE in KTRs. In a prospective cohort study of 592 healthy volunteers by Semba et al., fat mass was inversely associated with serum carboxymethyl lysine, a major circulating AGE, considering the possibility that AGE is stored in adipose tissue [28].

It is known that visceral fat is a protective factor in end-stage renal disease patients on maintenance HD, which is referred to as reverse epidemiology [60]. Moreover, it is well known that several traditional risk factors for CV disease in the general population, such as arterial hypertension, obesity, and hypercholesterolemia, are predictive of CV disease development in CKD, whereas a reverse effect is observed in those patients treated with dialysis. In dialysis patients, lower blood pressure, lower BMI, and lower serum cholesterol levels appear to be independent risk factors for CV disease. In some cases, a reversal back to traditional epidemiology has been described after successful KTX. More trials are needed to address whether modification of traditional and nontraditional CV risk factors can reduce the risk of CV disease in KTRs [61].

Because this is the first study to use the BIA method to analyze body composition and AGE in KTRs, and we have no other evidence to address this question, could it be that visceral fat favors the lower SAF AGE levels in KTRs? Considering the relatively small number of participants and all of the limitations resulting from the cross-sectional design of our study, we highlight an area that should be further investigated with a prospective study design in this population of patients.

Regarding adherence to the Mediterranean diet, the results of our final regression model showed no associations between MDSS score and AGE. However, adherence to specific foods and food groups showed associations with AGE in KTRs. For example, adherence to MeDi that recommended cereal intake at each meal showed an inverse association with AGE. The consumption of sweets two or fewer times per week, the consumption of red meat less than two times per week, and the daily consumption of nuts, as recommended by MeDi, showed reciprocal associations with AGE in Dalmatian KTRs. This is quite contradictory because red meat, sweets, and nuts are foods naturally rich in AGE [62]. It is important to note that we did not consider food preparation methods. For example, it has been studied that boiling or stewing meat results in more than twice the reduction in AGE levels than broiling the same piece of meat [62]. DeChristopher’s review suggests that the source of elevated N-3 carboxymethyl lysine, a well-studied marker of AGE formation, may not be dietary but rather intestinal [63].

The results of our logistic/linear regression presented in Table S1 show associations between the phase angle and the duration of dialysis before KTX with AGE. The phase angle is known to be a predictor of sarcopenia [64] and mortality in KTRs [65], and AGE, together with malnutrition, is a predictor of mortality in dialysis patients [50]. In a study by Hartog et al. on a KTR population, the duration of pre-transplant dialysis was also associated with AGE measured by SAF [54]. These findings may suggest an interplay between oxidative stress, sarcopenia, and the duration of dialysis treatment in KTRs, but further studies in a prospective design are needed to better explain this issue.

This study has some limitations, primarily resulting from the cross-sectional design, which prevented us from detecting causal associations. In addition, we included a relatively small but a representative number of participants because of the specificity of the target population. We did not consider food preparation type, although we used a validated MDSS questionnaire that considers food type but not portion sizes and food preparation type. Another limitation regarding the MDSS questionnaire could be a recall bias together with a low percentage of MeDi adherent KTRs, and regarding measurements with the AGE reader, although it is a validated device and an experienced investigator performed the measurements for three consecutive times, measurement error could have happened. We did not take physical activity into consideration when conducting this study, unfortunately. The strengths of our study are the representativeness of the study population and the innovative study objective for this population of patients.

## 5. Conclusions

In conclusion, the results of this cross-sectional study showed a high proportion of KTRs with high CV risk determined by AGE measured by SAF. Furthermore, the duration and type of dialysis treatment prior to KTX, the history of CV disease and the presence of CKD, together with the inflammation and nitrogen excretion parameters, showed significant associations with AGE in KTRs. Additionally, we also found that elements of nutritional status and MeDi adherence were important predictors for increased levels of AGE in this patient population. Finally, studies with a larger number of participants in a prospective design should be designed to investigate other potential factors contributing to AGE levels in KTRs.

This is the first study to elaborate on the dietary pattern, body composition, and AGE in KTRs with a focus on CV risk according to AGE value, which introduces additional knowledge into a field of untraditional CV risk factors and demonstrates a direction for adequate nutritional care in KTRs. Lifestyle modifications in KTRs should be considered for further investigation and clinical practice.

## Figures and Tables

**Table 1:** General characteristics of study participants and differences according to cardiovascular risk classification.

	Total (N = 85)	Mild to Moderate CV Risk Based on AGE (N = 26)	Severe CV Risk Based on AGE (N = 59)	*p* *
Age (years), median (IQR)	63 (15)	54 (28.5)	67 (12)	<0.001
Sex, N (%)				
Women	35 (41.18)	12 (46.15)	23 (38.98)	0.704
Men	50 (58.82)	14 (53.85)	36 (61.02)	
AGE, median (IQR)	3.3 (1.2)	2.6 (0.28)	3.6 (0.95)	<0.001
Time since kidney transplantation (years), median (IQR)	8 (9)	4.5 (8.75)	8 (10.5)	0.109
Dialysis duration (years), median (IQR)	2 (3.75)	2 (1.98)	2.5 (4.69)	0.082
Dialysis type, N (%)				
PD	27 (31.76)	15 (57.69)	12 (20.34)	0.003
HD	43 (50.59)	8 (30.77)	35 (59.32)	
PD + HD	15 (17.65)	3 (11.54)	12 (20.34)	
Smoking status, N (%)				
Never	35 (41.18)	14 (63.64)	21 (52.50)	0.353
Ex	15 (17.65)	3 (13.64)	12 (30.00)	
Current	12 (14.11)	5 (22.73)	7 (17.50)	
COMORBIDITIES				
Presence of arterial hypertension, N (%)				
No	5 (5.88)	2 (7.69)	3 (5.08)	1.000
Yes	80 (94.12)	24 (92.31)	56 (94.92)	
Presence of diabetes mellitus, N (%)				
No	65 (76.47)	22 (84.62)	43 (72.88)	0.369
Yes	20 (23.53)	4 (15.38)	16 (27.12)	
Presence of chronic kidney disease, N (%)				
eGFR > 60 mL/min/1.73 m^2^	25 (29.41)	14 (53.85)	11 (18.64)	0.002
eGFR < 60 mL/min/1.73 m^2^	60 (70.59)	12 (46.15)	48 (81.36)	
Presence of CVD, N (%)				
No	60 (70.59)	21 (80.77)	39 (66.1)	0.267
Yes	25 (29.41)	5 (19.23)	20 (33.9)	
Presence of CVA, N (%)				
No	74 (87.06)	23 (88.46)	51 (86.44)	1.000
Yes	11 (12.94)	3 (11.54)	8 (13.56)	
LABORATORY PARAMETERS				
Alb (g/L), mean (SD)	41.76 (2.93)	42.18 (2.77)	41.59 (3)	0.420
Ca (mmol/L), median (IQR)	2.42 (0.16)	2.41 (0.15)	2.42 (0.17)	0.682
CRP (mg/L), median (IQR)	2.15 (4)	1.55 (2.67)	2.65 (4.42)	0.243
E, mean (SD)	4.69 (0.69)	4.7 (0.72)	4.69 (0.68)	0.987
Glucose (mmol/L), median (IQR)	5.2 (0.9)	5.15 (0.95)	5.3 (1)	0.834
Hb (g/L), median (IQR)	132.38 (16.65)	132.35 (16.19)	132.39 (16.98)	0.991
K (mmol/L), median (IQR)	4.1 (0.6)	4.1 (0.5)	4 (0.7)	0.924
Total cholesterol (mmol/L), mean (SD)	5.39 (1.06)	5.39 (1.17)	5.39 (1.02)	0.992
Creatinine (mmol/L), median (IQR)	131 (52)	115.5 (61.5)	135 (46.5)	0.063
LDL cholesterol (mmol/L), mean (SD)	3.13 (0.95)	3.05 (1.08)	3.16 (0.9)	0.672
MCV (fL), median (IQR)	87.25 (7.1)	87.2 (6.9)	87.3 (7.15)	0.765
Na (mmol/L), median (IQR)	140.7 (2.44)	140.32 (2.01)	140.87 (2.61)	0.355
P (mmol/L), median (IQR)	1.03 (0.23)	1.04 (0.24)	1 (0.2)	0.264
Tgl (mmol/L), median (IQR)	1.8 (1.1)	1.9 (1)	1.71 (1.07)	0.370
Uric acid (mmol/L), mean (SD)	390.34 (91.2)	377.12 (100.86)	396.03 (87.02)	0.389
Urea (mmol/L), median (IQR)	9.7 (5.7)	8 (3.47)	10.8 (4.85)	0.002
eGFR (ml/min/1.73 m^2^), median (IQR)	43 (28.6)	61.95 (34.43)	39.7 (16.55)	0.009

* *p*-values were obtained with chi-square test for categorical data, *t*-test for parametric numerical data and Mann-Whitney U test for non-parametric numerical data Abbreviations: CV—cardiovascular, PD—peritoneal dialysis, HD—hemodialysis, AGE—advanced glycation end products, eGFR—estimated glomerular filtration rate using CKD-EPI (mL/min/1.73 m^2^), CVD—cardiovascular disease, CVA—cerebrovascular disease, Alb—serum albumin (g/L), Ca—calcium (mmol/L), CRP—C-reactive protein (mg/L), E—erythrocyte count, Hb—haemoglobin (g/L), K—potassium (mmol/l), LDL—low-density lipoprotein cholesterol (mmol/L), MCV—mean cellular volume (fL), Na—sodium (mmol/L), P—phosphates (mmol/L), Tgl—triglycerides (mmol/L), IQR- interquartile range, SD- standard deviation, N- number.

**Table 2:** Nutritional status and adherence to the Mediterranean diet of study participants and differences according to cardiovascular risk classification.

	Total (N = 85)	Mild to Moderate CV Risk Based on AGE (N = 26)	Severe CV Risk Based on AGE (N = 59)	*p* *
ANTHROPOMETRIC PARAMETERS				
Height (cm), mean (SD)	174.2 (9.99)	174.92 (10.81)	173.89 (9.71)	0.677
Weight (kg), mean (SD)	81.26 (15.59)	84.31 (17.34)	79.98 (14.76)	0.256
BMI (kg/m^2^), mean (SD)	26.68 (4.09)	27.52 (4.76)	26.33 (3.76)	0.236
BMI (kg/m^2^) as categories, N (%)				
<25, normal weight	28 (34.57)	8 (33.33)	20 (35.09)	0.114
25–30, overweight	37 (45.68)	8 (33.33)	29 (50.88)	
>30, obese	16 (19.75)	8 (33.33)	8 (14.04)	
Middle upper arm circumference (cm), median (IQR)	31 (5.25)	31 (3)	31 (5.5)	0.705
Waist circumference (cm), mean (SD)	99.48 (12.1)	99.9 (13.77)	99.31 (11.52)	0.851
WHtR, mean (SD)	0.57 (0.07)	0.57 (0.07)	0.57 (0.07)	0.916
BODY COMPOSITION				
Fat mass (kg), mean (SD)	20.5 (8.71)	23.36 (8.59)	19.28 (8.54)	0.054
Fat mass (%), mean (SD)	24.7 (8.87)	27.31 (7.27)	23.59 (9.31)	0.086
Fat-free mass (kg), mean (SD)	60.96 (12)	60.96 (12.35)	60.96 (11.96)	1.000
Visceral fat (level), mean (SD)	9.83 (3.85)	9 (4.4)	10.2 (3.55)	0.204
Metabolic age (year), mean (SD)	51.14 (13.16)	46.38 (16.07)	53.18 (11.25)	0.033
Muscle mass (kg), mean (SD)	57.92 (11.44)	57.92 (11.77)	57.92 (11.4)	0.998
Skeletal muscle mass (kg), median (IQR)	31.8 (12)	30.5 (12.92)	32.1 (11.67)	0.975
Skeletal muscle mass (%), median (IQR)	40.26 (6.29)	38.82 (5.22)	40.88 (6.64)	0.182
Phase angle (°), median (IQR)	5.09 (0.75)	5.36 (0.67)	4.98 (0.76)	0.036
Trunk fat mass (kg), median (IQR)	10.45 (5.72)	12.55 (5.85)	10.3 (5.45)	0.067
Mediterranean Diet Serving Score (MDSS)				
Total MDSS points, median (IQR)	8 (4)	8.5 (3)	8 (5.5)	0.882
Adherence to MeDi, N (%)				
MDSS < 14 points	75 (88.24)	24 (92.31)	51 (86.44)	0.683
MDSS ≥ 14 points	10 (11.76)	2 (7.69)	8 (13.56)	
Adherence to specific food or food group				
Fruits (no), N (%)	56 (65.88)	18 (69.23)	38 (64.41)	0.854
Fruits (yes), N (%)	29 (34.12)	8 (30.77)	21 (35.59)	
Vegetable (no), N (%)	68 (80)	22 (84.62)	46 (77.97)	0.680
Vegetable (yes), N (%)	17 (20)	4 (15.38)	13 (22.03)	
Cereals (no), N (%)	45 (52.94)	10 (38.46)	35 (59.32)	0.124
Cereals (yes), N (%)	40 (47.06)	16 (61.54)	24 (40.68)	
Potato (no), N (%)	18 (21.18)	4 (15.38)	14 (23.73)	0.562
Potato (yes), N (%)	67 (78.82)	22 (84.62)	45 (76.27)	
Olive oil (no), N (%)	60 (70.59)	17 (65.38)	43 (72.88)	0.659
Olive oil (yes), N (%)	25 (29.41)	9 (34.62)	16 (27.12)	
Nuts (no), N (%)	72 (84.71)	24 (92.31)	48 (81.36)	0.334
Nuts (yes), N (%)	13 (15.29)	2 (7.69)	11 (18.64)	
Dairy (no), N (%)	28 (32.94)	10 (38.46)	18 (30.51)	0.639
Dairy (yes), N (%)	57 (67.06)	16 (61.54)	41 (69.49)	
Beans (no), N (%)	57 (67.06)	16 (61.54)	41 (69.49)	0.639
Beans (yes), N (%)	28 (32.94)	10 (38.46)	18 (30.51)	
Eggs (no), N (%)	57 (67.06)	16 (61.54)	41 (69.49)	0.639
Eggs (yes), N (%)	28 (32.94)	10 (38.46)	18 (30.51)	
Fish (no), N (%)	50 (58.82)	15 (57.69)	35 (59.32)	1.000
Fish (yes), N (%)	35 (41.18)	11 (42.31)	24 (40.68)	
White meat (no), N (%)	59 (69.41)	16 (61.54)	43 (72.88)	0.429
White meat (yes), N (%)	26 (30.59)	10 (38.46)	16 (27.12)	
Red meat (no), N (%)	52 (61.18)	19 (73.08)	33 (55.93)	0.210
Red meat (yes), N (%)	33 (38.82)	7 (26.92)	26 (44.07)	
Sweets (no), N (%)	37 (43.53)	17 (65.38)	20 (33.9)	0.014
Sweets (yes), N (%)	48 (56.47)	9 (34.62)	39 (66.1)	
Alcohol (no), N (%)	71 (83.53)	22 (84.62)	49 (83.05)	1.000
Alcohol (yes), N (%)	14 (16.47)	4 (15.38)	10 (16.95)	
Consumption frequency of each food or food group **				
Fruits, median (IQR)	2 (1)	2 (1)	2 (1)	0.653
Vegetable, median (IQR)	2 (1)	2 (1)	2 (1)	0.689
Cereals, median (IQR)	2 (1)	1 (1)	2 (1)	0.186
Potato, median (IQR)	3 (1)	3 (1)	3 (1)	0.931
Olive oil, median (IQR)	2 (2)	2 (2)	2 (2)	0.794
Nuts, median (IQR)	5 (3)	4 (2.75)	5 (3)	0.368
Dairy, median (IQR)	2 (1)	2 (1)	2 (1)	0.485
Beans, median (IQR)	5 (1)	5 (1.75)	5 (1)	0.374
Eggs, median (IQR)	5 (1)	5 (1)	5 (1)	0.365
Fish, median (IQR)	5 (1)	5 (1)	5 (1)	0.808
White meat, median (IQR)	4 (1)	4 (1)	4 (2)	0.623
Red meat, median (IQR)	4 (2)	4 (1.75)	4 (2)	0.184
Sweets, median (IQR)	4 (4)	3 (3)	5 (3)	0.018
Alcohol, median (IQR)	7 (2.5)	6.5 (2)	7 (3)	0.215

* *p*-values were obtained with the chi-square test for categorical data, *t*-test for parametric numerical data, and Mann-Whitney U test for non-parametric numerical data. ** Consumption frequency ranged from 1 to 7 where 1 denotes “consumed with every meal”, while 7 denotes “consumed rarely or never”. Abbreviations: AGE—advanced glycation end products, CV—cardiovascular, BMI—Body Mass Index (kg/m^2^), WHtR—waist-to-height ratio, MDSS—Mediterranean Diet Serving Score, IQR- interquartile range, SD- standard deviation, N- number.

**Table 3:** Linear and logistic LASSO regression models to assess the predictors for severe CV risk based on AGE.

	LASSO Regression (Severe vs. Mild to Moderate CV Risk Based on AGE)				LASSO Regression with AGE			
	Coefficient	Lower CI	Upper CI	*p* *	Coefficient	Lower CI	Upper CI	*p* *
Age (years)	0.098	0.041	0.260	0.001	0.018	0.005	0.032	0.005
Dialysis (type)	1.621	−2.210	2.418	0.273	0.095	−0.45	0.322	0.411
Dialysis (years)	/				0.030	−0.036	0.074	0.171
Comorbidities								
CV disease (yes)	/				0.154	−0.629	0.493	0.383
CKD (yes)	1.670	0.403	40.596	0.017	0.435	0.028	0.799	0.018
Laboratory parameters								
Ca (mmol/L)	−1.322	−27.967	17.838	0.371	/			
CRP (mg/L)	0.075	−2.912	0.370	0.828	0.029	−0.021	0.064	0.113
P (mmol/L)	/				−0.293	−1.039	1.889	0.479
Uric acid (mmol/L)	/				0.002	0	0.004	0.008
Urea (mmol/L)	0.002	−7.341	0.116	0.950	/			
Body composition parameters								
Skeletal muscle mass (%)	0.092	−0.348	1.033	0.221	/			
Trunk fat mass (kg)	−0.021	−0.942	0.798	0.451	−0.046	−0.075	−0.015	0.002
Mediterranean diet								
Cereals (yes)	−1.017	−2.528	1.646	0.218	/			
Nuts (yes)	/				0.454	−0.028	0.866	0.031
Red meat (yes)	0.851	−4.441	1.983	0.490	/			
Sweets (yes)	0.926	−1.365	9.593	0.112	/			

* *p*-values were obtained with post-selection inference for LASSO regression models. Abbreviations: AGE—advanced glycation end products, CV—cardiovascular, CKD—chronic kidney disease (estimated glomerular filtration rate (eGFR) using CKD-EPI < 60 mL/min/1.73 m^2^), Ca—calcium (mmol/L), CRP—C-reactive protein (mg/L), P—phosphates (mmol/L), CI- confidence interval.

## Data Availability

Raw data are available at corresponding author mail: josiparadic1973@gmail.com.

## Supplementary Materials

The following supporting information can be downloaded at: https://www.mdpi.com/article/10.3390/ijerph191711060/s1, Figure S1: Correlation between trunk fat mass and years spent on dialysis in all KTRs; Figure S2: Correlation between trunk fat mass and years spent on dialysis in KTRs on HD; Figure S3: Correlation between trunk fat mass and years spent on dialysis in KTRs on HD and PD; Figure S4: Correlation between trunk fat mass and years after KTX in all KTRs; Figure S5: Correlation between trunk fat mass and years spent on dialysis in KTRs on PD; Figure S6: Correlation between trunk fat mass and years after KTX in KTRs on PD; Figure S7: Correlation between trunk fat mass and years after KTX in KTRs on HD; Figure S8: Correlation between trunk fat mass and years after KTX in KTRs on HD and PD; Table S1: Univariate linear and logistic regression of AGE in KTRs.
