# Supplementary material for: Associations between Advanced Glycation End Products, Body Composition and Mediterranean Diet Adherence in Kidney Transplant Recipients
Source: Int J Environ Res Public Health. 2022 Sep 4;19(17):11060. doi: 10.3390/ijerph191711060 (PMC9518364; doi:10.3390/ijerph191711060)
Supplement: Supplementary file 1 [file ijerph-19-11060-s001.zip › Table S1.pdf]

Table S1. Univariate linear and logistic regression of AGE in KTRs.

	Univariate logistic regression (severe vs mild to moderate CV risk based on AGE)			Univariate linear regression with AGE		
	**OR**	**95% CI**	**P**	**Beta**	**SE**	**P**
Age	1.09	1.05-1.14	0.000	0.033	0.006	0.000
Sex (men)	1.34	0.53-3.41	0.536	0.217	0.200	0.282
Transplantation yrs	1.07	0.98-1.16	0.140	0.011	0.017	0.524
Dialysis type (HD)	5.47	1.86-16.1	0.002	0.438	0.220	0.050
Dialysis type (PD+HD)	5.00	1.14-21.86	0.033	0.416	0.289	0.153
Dialysis yrs	1.17	0.98-1.41	0.085	0.068	0.025	0.007
Smoking status	1.1	0.57-2.14	0.778	-0.071	0.159	0.654
**COMORBIDITIES**						
AH (yes)	1.56	0.24-9.91	0.640	0.445	0.419	0.291
DM (yes)	2.05	0.61-6.86	0.246	0.342	0.231	0.143
CVD (yes)	2.15	0.71-6.56	0.177	0.648	0.206	0.002
CVA (yes)	1.20	0.29-4.95	0.798	0.001	0.296	0.996
eGFR cat (yes)	5.09	1.85-14	0.002	0.837	0.197	0.000
**LABORATORY PARAMETERS**						
Alb	0.93	0.79-1.1	0.416	-0.043	0.035	0.221
Ca	0.15	0.01-2.43	0.180	-0.355	0.483	0.464
CRP	1.11	0.96-1.28	0.146	0.069	0.021	0.002
E	0.99	0.5-1.97	0.987	0.034	0.146	0.815
GUP	1.01	0.67-1.5	0.979	0.014	0.086	0.867
Hb	1.00	0.97-1.03	0.991	0.000	0.006	0.941
K	1.21	0.43-3.4	0.721	-0.003	0.220	0.989
Cholesterol	1.00	0.61-1.62	0.992	-0.161	0.105	0.131
Creatinin	1.01	1-1.02	0.103	0.003	0.002	0.099
LDL	1.13	0.65-1.95	0.667	-0.112	0.117	0.344
MCV	1.01	0.94-1.1	0.747	0.006	0.017	0.724
Na	1.10	0.9-1.34	0.351	0.005	0.043	0.910
P	0.47	0.05-4.41	0.505	-0.780	0.484	0.111
Tgl	0.73	0.44-1.19	0.207	-0.085	0.108	0.430
Urate	1.00	1-1.01	0.385	0.003	0.001	0.023
Urea	1.20	1.04-1.38	0.010	0.062	0.020	0.003
eGF	0.97	0.94-0.99	0.006	-0.015	0.004	0.001
**ANTHROPOMETRIC PARAMETERS**						
Height	0.99	0.94-1.04	0.673	-0.008	0.010	0.420
Weight	0.98	0.95-1.01	0.255	-0.011	0.006	0.084
BMI	0.93	0.83-1.05	0.235	-0.041	0.025	0.103
Overweight	1.45	0.47-4.5	0.521	-0.042	0.230	0.854
Obese	0.40	0.11-1.44	0.160	-0.346	0.288	0.233
UpperArm circ.	1.01	0.95-1.07	0.729	-0.007	0.008	0.427
Waist circ.	1.00	0.96-1.04	0.849	-0.005	0.007	0.504
WHtR	1.51	0-2935.04	0.915	-0.274	1.268	0.829
**BODY COMPOSITION PARAMETERS**						

Fat mass (kg)	0.95	0.89-1	0.059	-0.020	0.012	0.086
Fat mass (%)	0.95	0.9-1.01	0.089	-0.018	0.012	0.135
Fat free mass (kg)	1.00	0.96-1.04	1.000	-0.003	0.009	0.715
Visceral fat	1.09	0.95-1.24	0.204	0.030	0.027	0.286
Metabolic age	1.04	1-1.08	0.040	0.013	0.008	0.112
Muscle mass (kg)	1.00	0.96-1.04	0.998	-0.003	0.009	0.715
Skeletal muscle mass (kg)	1.00	0.94-1.06	0.908	-0.004	0.013	0.747
Skeletal muscle mass (%)	1.06	0.97-1.14	0.182	0.019	0.016	0.257
Phase angle	0.50	0.26-0.97	0.041	-0.290	0.134	0.034
Trunk visceral fat	0.90	0.82-0.99	0.035	-0.059	0.019	0.003
**MEDITERRANEAN DIET**						
MDSS	1.00	0.88-1.14	0.999	0.005	0.028	0.849
MDSS cat (yes)	1.88	0.37-9.55	0.445	0.285	0.306	0.355
Fruits (yes)	1.24	0.46-3.34	0.666	0.180	0.208	0.391
Vegetables (yes)	1.55	0.45-5.32	0.482	0.090	0.248	0.718
Cereals (yes)	0.43	0.17-1.1	0.079	-0.196	0.198	0.324
Potato (yes)	0.58	0.17-1.98	0.389	-0.424	0.238	0.079
Olive oil (yes)	0.70	0.26-1.89	0.486	-0.106	0.217	0.627
Nuts (yes)	2.75	0.56-13.41	0.211	0.655	0.266	0.016
Dairy (yes)	1.42	0.54-3.74	0.473	0.083	0.211	0.693
Beans (yes)	0.70	0.27-1.84	0.473	0.082	0.211	0.700
Eggs (yes)	0.70	0.27-1.84	0.473	-0.052	0.211	0.808
Fish (yes)	0.94	0.37-2.38	0.888	-0.115	0.201	0.571
White meat (yes)	0.60	0.22-1.58	0.298	-0.192	0.214	0.372
Red meat (yes)	2.14	0.78-5.86	0.139	-0.048	0.203	0.816
Sweets (yes)	3.68	1.39-9.73	0.009	0.223	0.199	0.265
Alcohol (yes)	1.12	0.32-3.97	0.858	-0.007	0.267	0.979
Fruits freq	1.26	0.71-2.25	0.431	-0.049	0.119	0.681
Vegetables freq	1.06	0.66-1.7	0.817	0.025	0.102	0.807
Cereals freq	1.03	0.61-1.73	0.912	-0.083	0.111	0.453
Potato freq	0.99	0.7-1.4	0.953	-0.066	0.074	0.379
Olive oil freq	1.04	0.78-1.4	0.780	-0.030	0.062	0.626
Nuts freq	1.09	0.85-1.41	0.487	-0.052	0.055	0.347
Dairy freq	0.96	0.72-1.28	0.769	-0.046	0.063	0.467
Beans freq	1.26	0.88-1.81	0.211	0.035	0.078	0.657
Eggs freq	1.18	0.82-1.68	0.370	-0.006	0.077	0.937
Fish freq	0.97	0.65-1.44	0.865	-0.038	0.085	0.661
White meat freq	1.12	0.77-1.64	0.546	-0.030	0.081	0.715
Red meat freq	1.28	0.89-1.85	0.181	0.042	0.076	0.580
Sweets freq	1.36	1.05-1.75	0.018	0.025	0.050	0.616
Alcohol freq	1.09	0.86-1.38	0.478	0.013	0.052	0.804

Abbreviations: CV—cardiovascular, PD—peritoneal dialysis, HD—hemodialysis, AGE—advanced glycation end products, AH—arterial hypertension, DM—diabetes mellitus, CVD—cardiovascular disease, CVA—cerebrovascular disease, eGFR—estimated glomerular filtration rate using CKD-EPI (mL/min/1.73m^2), Alb—serum albumin (g/L), Ca—calcium (mmol/L), CRP—C-reactive protein (mg/L), E—erithrocyte count, GUP—glucose (mmol/L), Hb—haemoglobin (g/L), K—potassium (mmol/l), LDL—low-density lipoprotein cholesterol (mmol/L),

MCV—mean cellular volume (fL), Na—sodium (mmol/L), P—phosphates (mmol/L), Tgl—triglycerides (mmol/L), BMI—body mass index, circ—circumference, WHtR—waist to height ratio, MDSS—Mediterranean Diet Serving Score, freq- frequency ranged from 1 to 7 where 1 denotes "consumed w`ith every meal", while 7 denotes "consumed rarely or never", CI—confidence interval, SE—standard error, OR—odds ratio.
