# Supplementary figures and images for: Associations between Advanced Glycation End Products, Body Composition and Mediterranean Diet Adherence in Kidney Transplant Recipients
Source: Int J Environ Res Public Health. 2022 Sep 4;19(17):11060. doi: 10.3390/ijerph191711060 (PMC9518364; doi:10.3390/ijerph191711060)

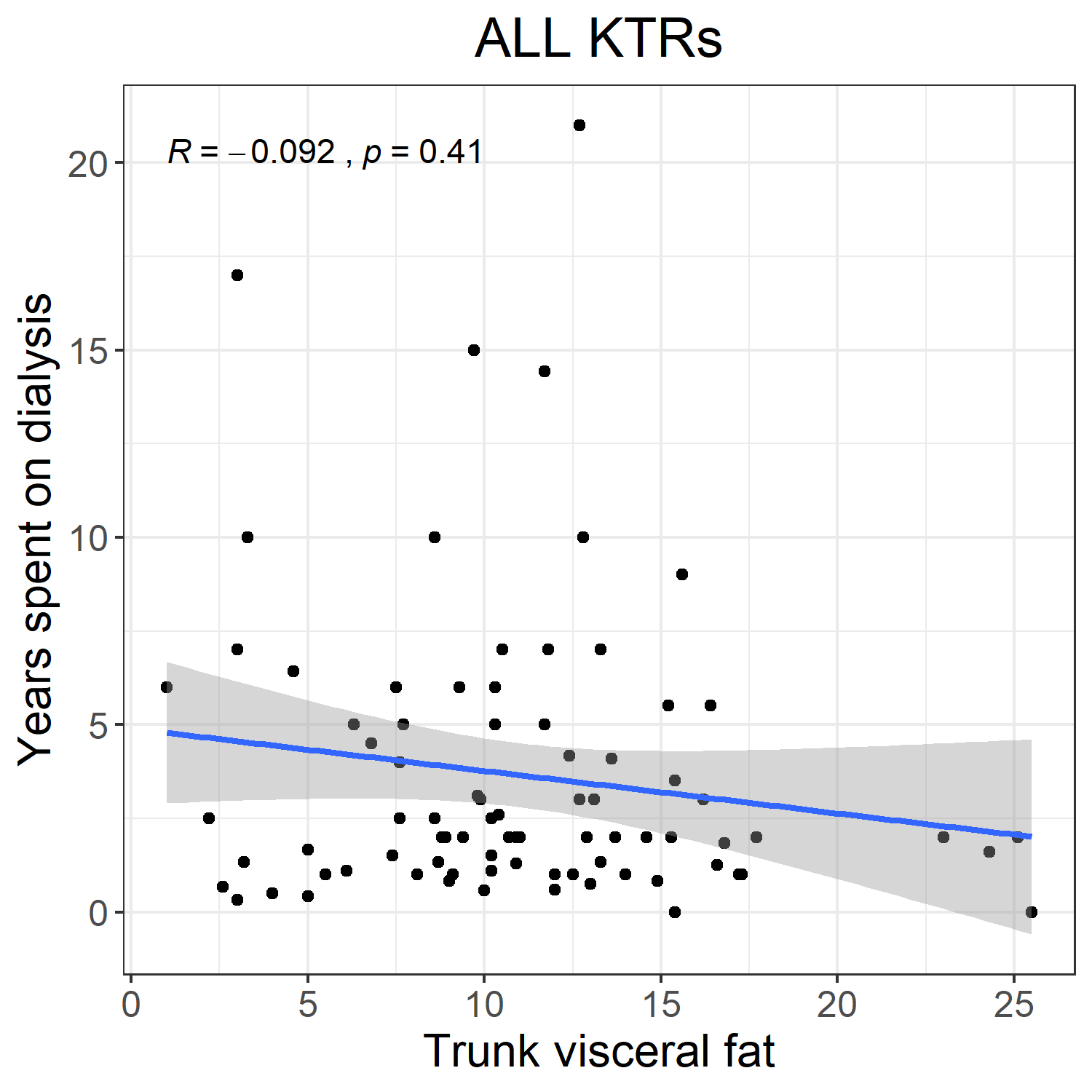

Supplement: Supplementary file 1 [file ijerph-19-11060-s001.zip › Figure S1.png]

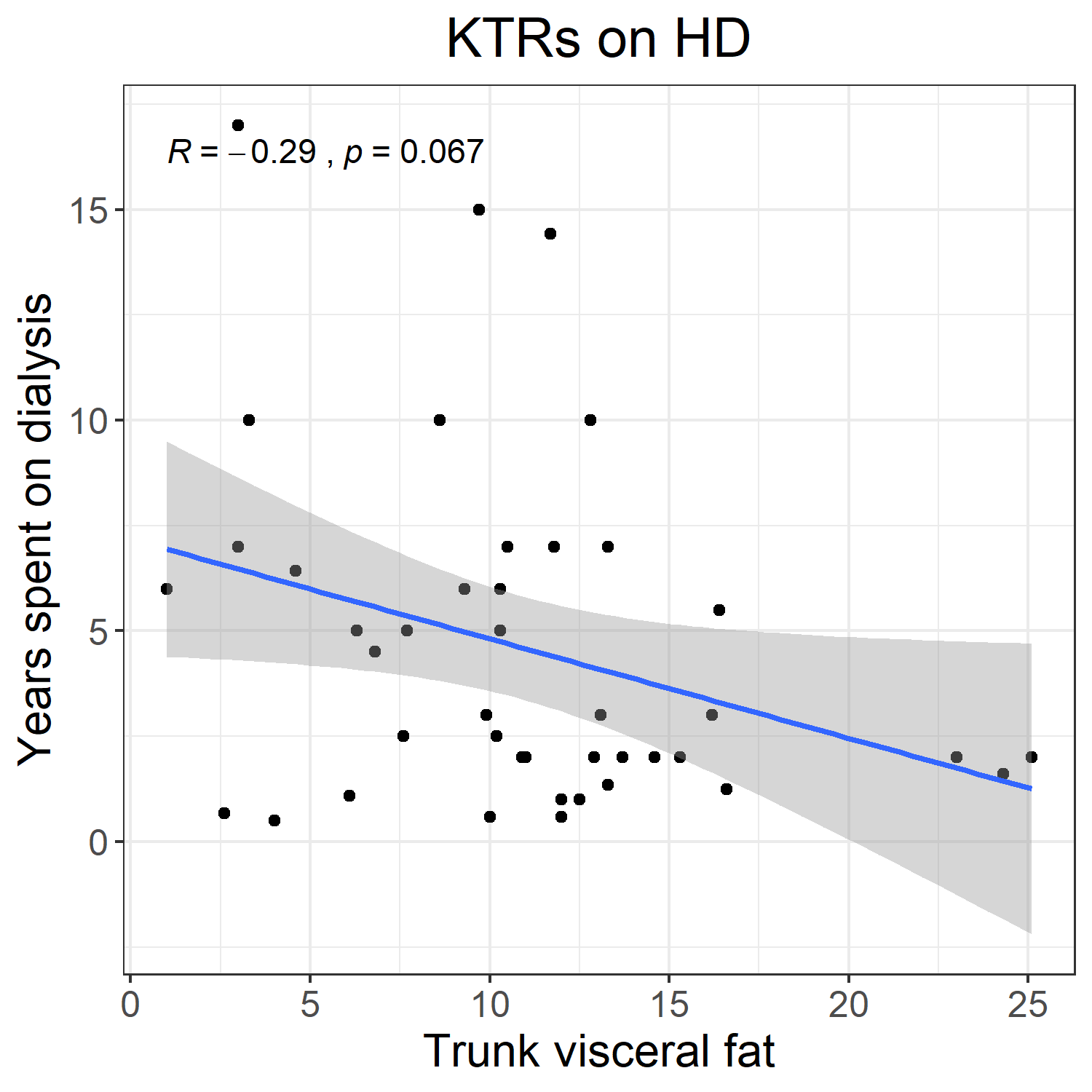

Supplement: Supplementary file 1 [file ijerph-19-11060-s001.zip › Figure S2.png]

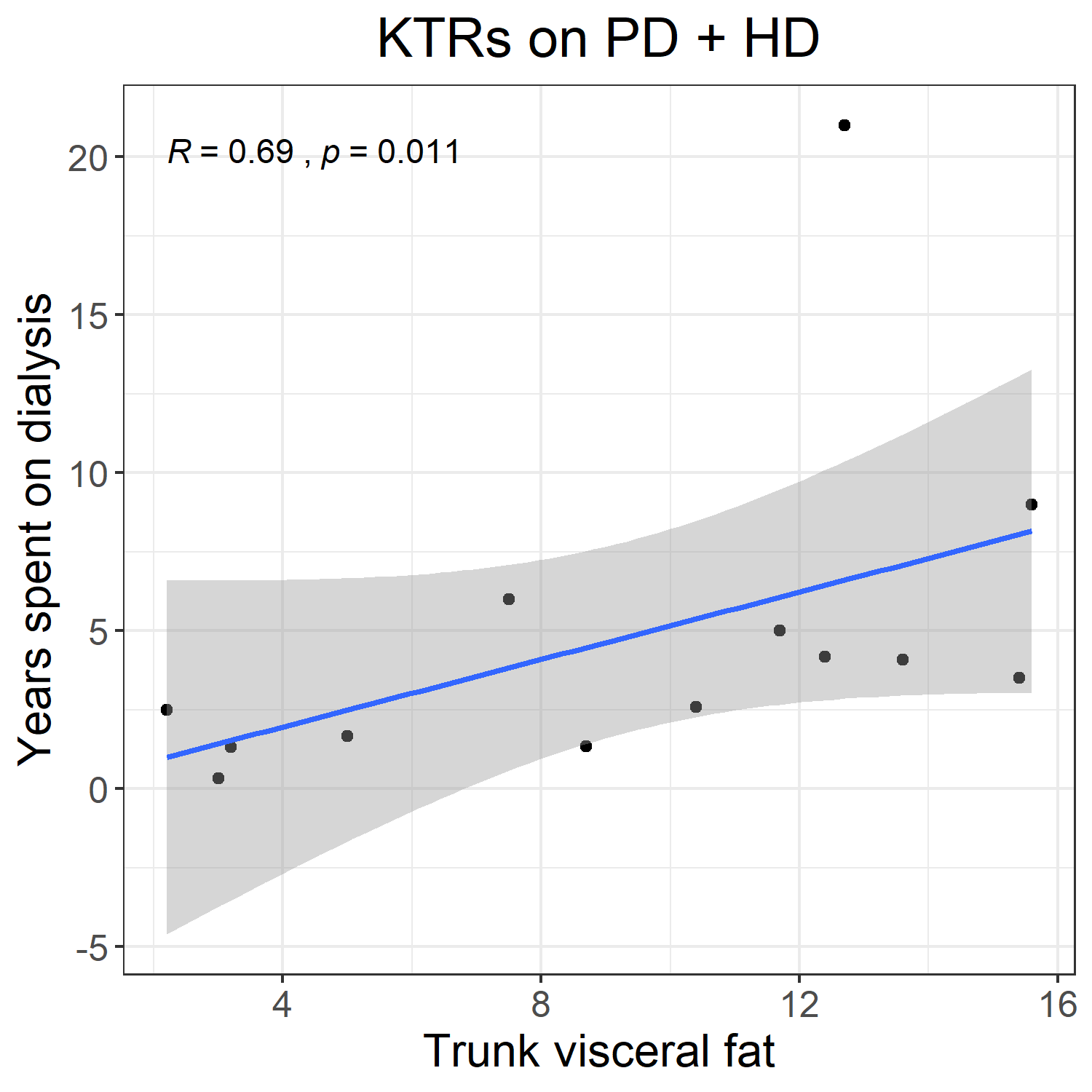

Supplement: Supplementary file 1 [file ijerph-19-11060-s001.zip › Figure S3.png]

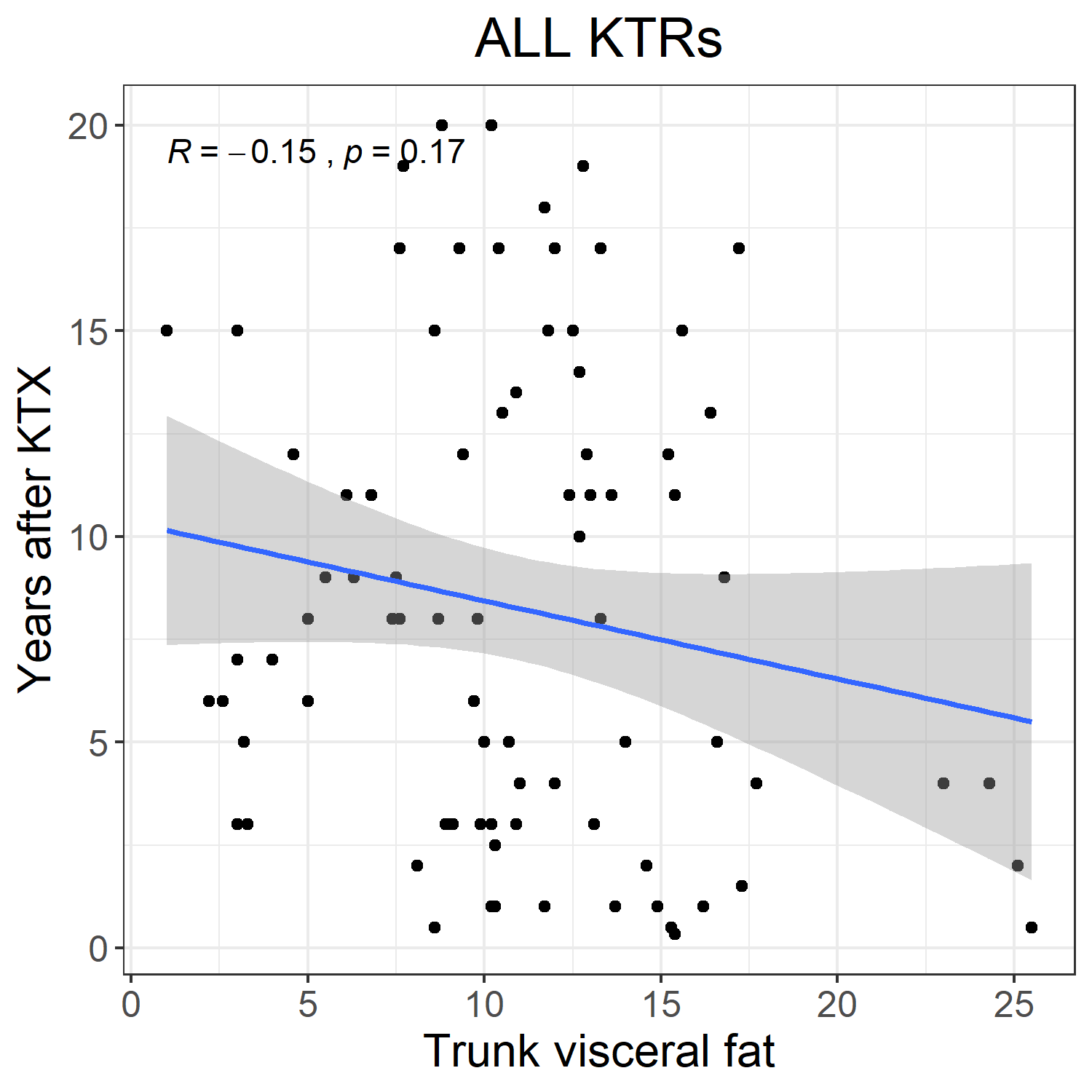

Supplement: Supplementary file 1 [file ijerph-19-11060-s001.zip › Figure S4.png]

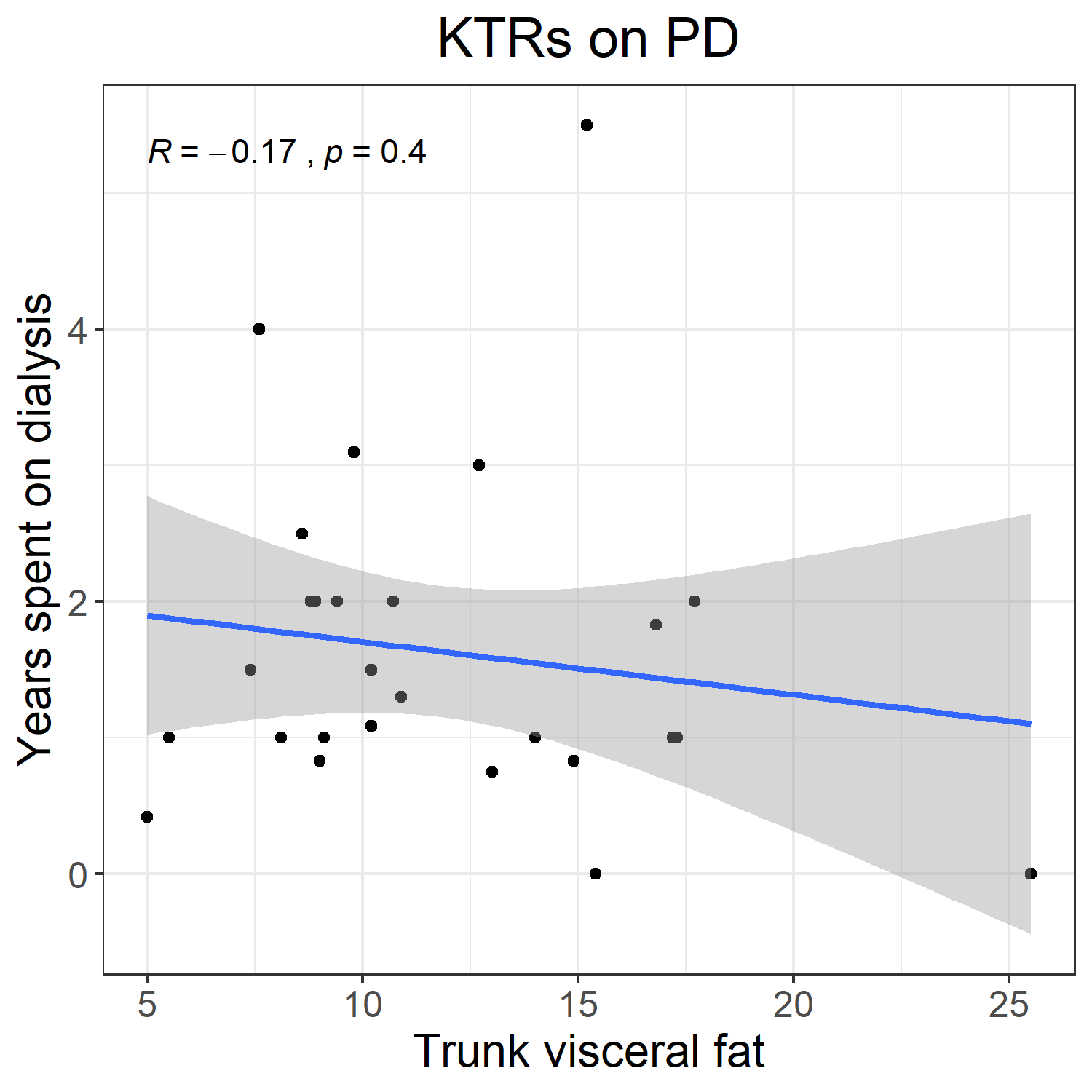

Supplement: Supplementary file 1 [file ijerph-19-11060-s001.zip › Figure S5.png]

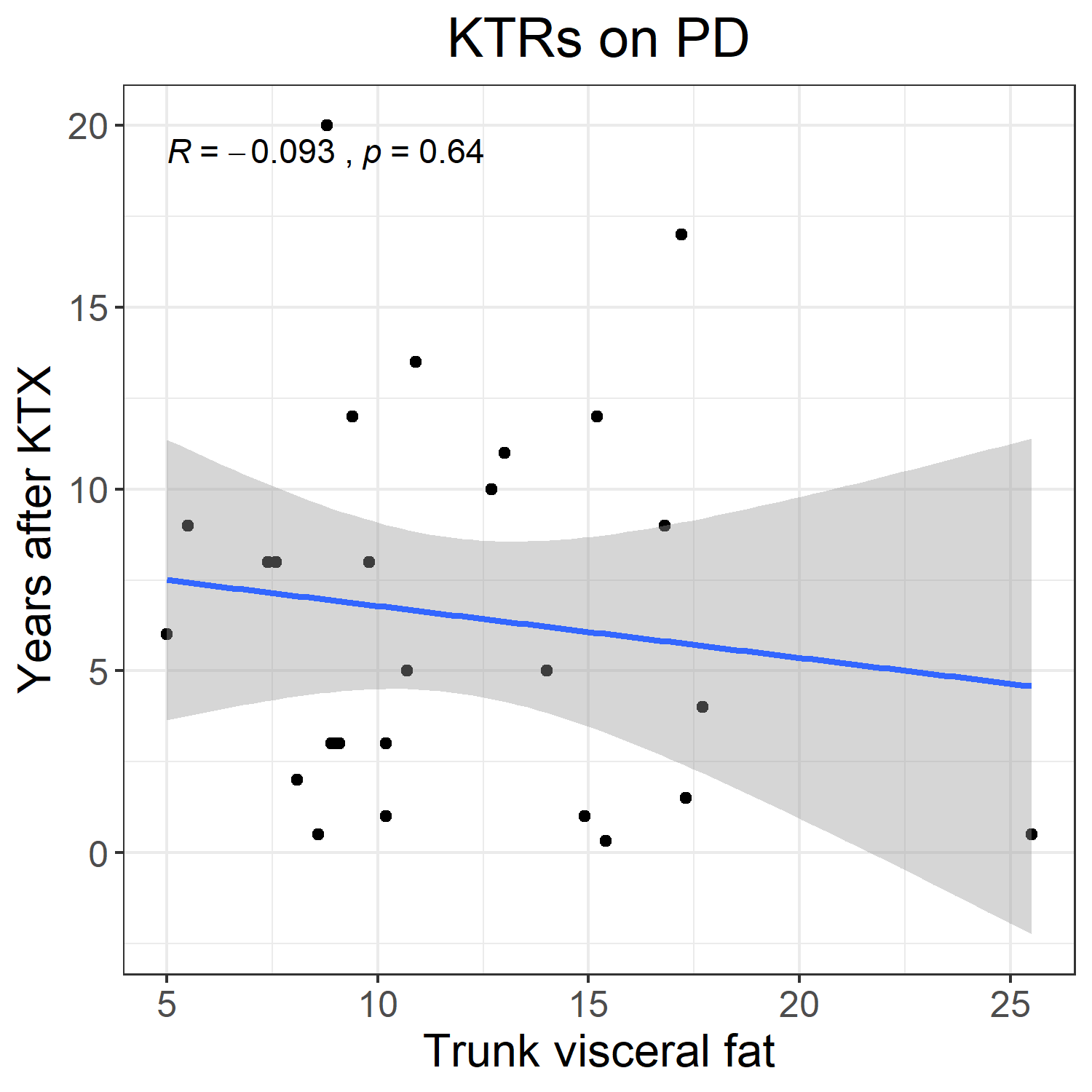

Supplement: Supplementary file 1 [file ijerph-19-11060-s001.zip › Figure S6.png]

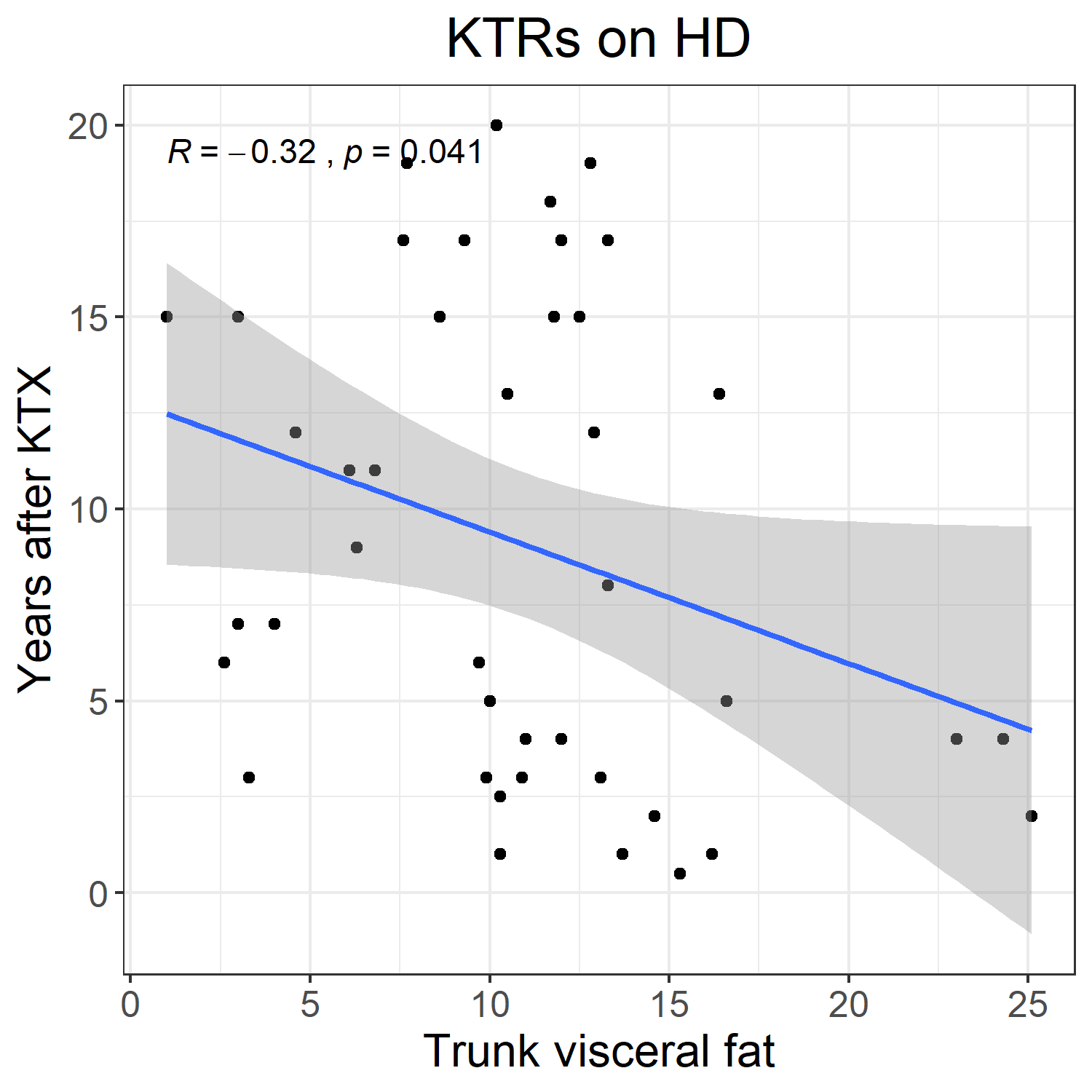

Supplement: Supplementary file 1 [file ijerph-19-11060-s001.zip › Figure S7.png]

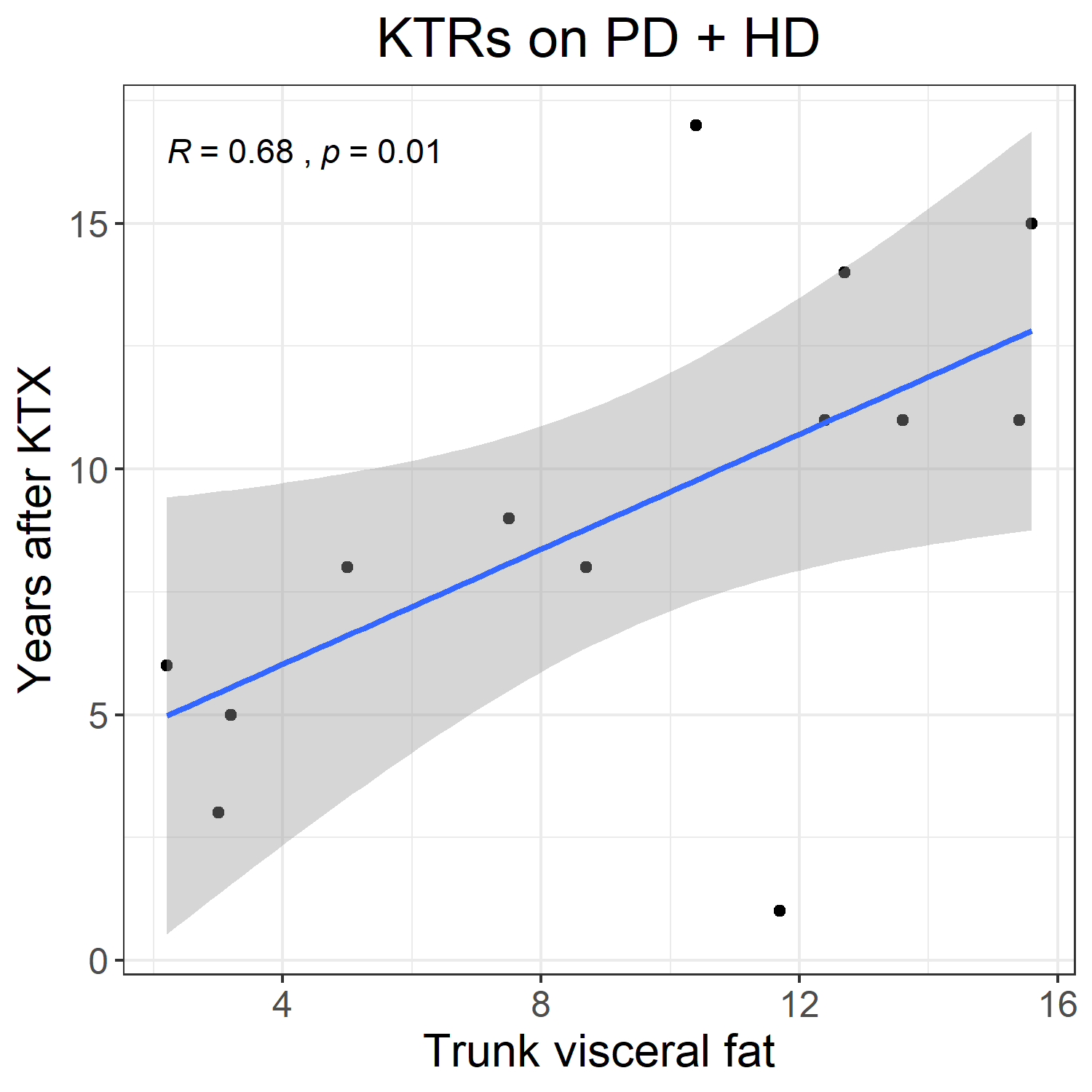

Supplement: Supplementary file 1 [file ijerph-19-11060-s001.zip › Figure S8.png]
